# Infections among Contacts of Patients with Nipah Virus, India

**DOI:** 10.3201/eid2608.190722

**Published:** 2020-08

**Authors:** Chong Tin Tan, Kum Thong Wong

**Affiliations:** University of Malaya, Kuala Lumpur, Malaysia

**Keywords:** viruses, zoonoses, respiratory infections, vector-borne infections, Nipah virus, Nipah virus fever, encephalitis, brain lesions, transmission, Nipah, seropositive, late-onset, India

**To the Editor:** Kumar et al. recently reported 3 asymptomatic, seropositive persons with Nipah virus (NiV) among 279 contacts of 18 NiV-infected patients (*1*). In the 1998–1999 NiV outbreak in Malaysia, asymptomatic, seropositive persons, most of whom were farm workers and soldiers involved in pig culling, also were identified (*2*,*3*). Furthermore, some additional cases might not have been detected among patients who had mild, nonencephalitic symptoms (e.g., fever, influenza-like illness). Up to 16% of asymptomatic, seropositive persons exhibited lesions in their brain magnetic resonance imaging (MRI) scans, albeit a slightly smaller number than in patients with acute NiV encephalitis (*2*). These discrete, small high-signal lesions in the cerebrum were best seen on fluid-attenuated inversion recovery images (*2*). In another study, a seropositive nurse who was exposed only to patients also had similar brain lesions visible on an MRI scan, suggesting person-to-person transmission (*4*). Whether late-onset NiV encephalitis (i.e., encephalitis in an asymptomatic or mildly symptomatic person) would develop in any of these persons, especially those with brain lesions visible on MRI scan, is unknown. In another cohort, approximately 5% of asymptomatic or mildly symptomatic persons (*5*) had febrile encephalitis characterized by headache, seizures, focal neurologic signs, and cerebrospinal fluid pleocytosis, a set of symptoms and signs distinct from acute NiV encephalitis. In patients who survived acute NiV encephalitis, a clinicopathologically similar relapsing encephalitis has also been reported (*5*). The death rate was ≈20% during the clinical course of late-onset/relapsing NiV encephalitis (5).

Physicians should consider using brain MRI to identify subclinical brain lesions in asymptomatic, seropositive Nipah patients. Moreover, physicians should advise patients of the possibility that late-onset encephalitis might occur months or even years after virus exposure (*5*). New treatments are in development or on trial, so patients with these complications may be offered access to effective treatments.
